# Perceptions of mindfulness to pregnant women with gestational diabetes: an exploratory qualitative Portuguese study

**DOI:** 10.3389/fgwh.2025.1558231

**Published:** 2025-09-11

**Authors:** Sandra Seixinho, Maria Helena Presado

**Affiliations:** Nursing Research, Innovation and Development Centre of Lisbon (CIDNUR), Escola Superior de Enfermagem de Lisboa, Lisbon, Portugal

**Keywords:** mindfulness, gestational diabetes, pregnant women, high-risk pregnancy, perceptions

## Abstract

**Introduction:**

Gestational diabetes (GD) significantly impacts maternal well-being, influencing both physical and psychological health. Non-pharmacological interventions, such as mindfulness, have emerged as potential nursing strategies to promote positive experiences during pregnancy. This study investigates the perceptions of mindfulness in pregnant women diagnosed with GD, with the aim of analyzing the perceptions of pregnant women about mindfulness. The research question was defined as “What are the perceptions of mindfulness to diabetic pregnant women?”.

**Methods:**

An exploratory qualitative approach was employed to deeply explore the perceptions of pregnant women regarding mindfulness. Seven pregnant women with GD, who met specific inclusion criteria, were intentionally selected and invited to participate in the study. Data were collected through semi-structured interviews and analyzed using Bardin's content analysis method.

**Results:**

Participants perceived mindfulness as beneficial in several areas. Improvements in general well-being, physical and psychological health, better interpersonal relationships, increased relaxation, greater preparation for childbirth, and strengthened confidence during the transition to motherhood.

**Discussion:**

The results indicate that mindfulness is perceived by pregnant women with GD as a valuable tool for improve their well-being and facilitating a smoother transition to motherhood. The study highlights the importance of incorporating mindfulness into nurse care, especially in the context of high-risk pregnancies. The results suggest that mindfulness can be integrated into nursing practices to more effectively support pregnant women. Further research is recommended to explore the broader implications of mindfulness in high-risk pregnancy care.

## 1 Introduction

Pregnancy is accompanied by significant physiological, psychological, and social changes (1), which can include increased anxiety and stress, with negative consequences for both the pregnant woman and the fetus (2). The World Health Organization (3) recommends non-pharmacological interventions as a first-line approach, particularly for the management of stress and pain.

Among these interventions, mindfulness and yoga have gained attention for their therapeutic potential during pregnancy. A study by Abirami and Judie (4) demonstrated a statistically significant improvement (*p* ≤ 0.001) in maternal, fetal, and neonatal outcomes among women with gestational diabetes (GD) who practiced yoga compared to a control group. The authors concluded that yoga should be considered an adjuvant therapy for pregnant women with GD to prevent complications and improve maternal and fetal outcomes.

Mindfulness is a non-pharmacological intervention based on meditative practices (5, 6). It aims to increase awareness through breathing exercises and heightened attention to bodily sensations, thoughts, emotions, and physical experiences. Mindfulness practices emphasize core attitudes such as non-judgment, patience, trust, non-striving, acceptance, generosity, gratitude, compassion, kindness, and tolerance (7).

Although studies specifically focused on mindfulness during pregnancy are limited, substantial evidence supports the benefits of mindfulness interventions outside the perinatal context (8). Research indicates that mindfulness practice reduces stress, anxiety, and depression (9–14). Furthermore, mindfulness fosters maternal-fetal bonding, enhances maternal responsiveness to the infant's needs postpartum (11), improves infant behavior (15, 16), and may serve as an alternative or complementary approach to pharmacotherapy for high-risk pregnancies affected by psychiatric disorders (17).

Mindfulness interventions can also contribute to improvements in maternal mental health, with studies reporting reductions in anxiety, enhancements in self-compassion and compassion (11), significant increases in mindfulness skills (14), and improved stress management during pregnancy (18).

The psychological effects of mindfulness appear particularly prominent. Studies report reductions in depressive symptoms, anxiety, stress, and fear of childbirth, along with improvements in perinatal mental health and increased maternal self-awareness (16). Among women with high-risk pregnancies, where depression and anxiety prevalence is elevated, mindfulness interventions have been associated with increased well-being and reduced anxiety (12). Mindfulness may also reduce the risk of preterm birth by mitigating anxiety levels (19).

However, mindfulness-based therapies remain relatively understudied in the context of preventing perinatal depression, although preliminary evidence suggests that they may benefit pregnant women with a history of depression (20, 21). Hicks et al. (22) also highlights the potential of mindfulness interventions in treating prenatal depression.

In the context of diabetes, mindfulness has been associated with prevention of glucose intolerance and a reduction in opioid use during labor (15, 16).

While there is substantial scientific evidence supporting the benefits of mindfulness in the general population, specific research focusing on pregnant women remains limited (23). Given that most reported benefits are psychological, promoting the mental health of pregnant women is crucial to enhance their adaptive capacity during pregnancy, a period associated with increased vulnerability to risk factors (24). Nevertheless, current evidence remains insufficient to formally recommend mindfulness as a standard intervention for the promotion of perinatal mental health (25).

## 2 Materials and methods

This study adhered to the Standards for Reporting Qualitative Research (SRQR) as proposed by O'Brien et al. (26) to ensure transparency and rigor in the reporting of qualitative research. The SRQR comprises 21 criteria that guide the comprehensive and accurate presentation of qualitative studies. A detailed application of these standards to the present study was undertaken.

The primary researcher is a nurse with professional experience in supporting pregnant women, including in the application of mindfulness-based interventions. During the study, the researcher maintained a professional relationship with the participants but did not provide direct clinical care, although familiarity with the institutional context was present. To enhance the trustworthiness of the study, reflexive practices were adopted. The primary researcher's familiarity with mindfulness practices, while beneficial in understanding participants' experiences, may have introduced interpretive bias. To mitigate this, a reflexive journal was maintained to record assumptions, emotional reactions, and analytic decisions throughout the research process. This strategy supported ongoing self-awareness and critical reflection. Moreover, the use of a semi-structured interview guide, detailed informed consent procedures, and manual coding strategies contributed to minimizing potential bias and ensuring rigor in both data collection and analysis.

### 2.1 Participants

#### 2.1.1 Participant inclusion and exclusion criteria

Given the nature of the research, the essential characteristics for study inclusion (27), and the research question and objectives (28), the following inclusion criteria were established:

- Pregnant women aged 18 years or older.
- Diagnosis of gestational diabetes (GD).
- Under follow-up care at the diabetes clinic during the study period.
- Agreed to participate in the study.

The decision to focus on pregnant women with GD was based on the prevalence of this condition during pregnancy. Pregnant women who did not feel comfortable practicing mindfulness, whose clinical condition prevented participation, or who experienced communication difficulties, as well as those whose condition was exacerbated by the disease, were excluded from the study.

#### 2.1.2 Participant selection

The selection of participants in this study involved intentional sampling of a group of pregnant women who were readily accessible through the hospital's prenatal care services. This purposive sample was designed to select participants most likely to provide rich, relevant, and diverse insights into the phenomenon under study, in accordance with qualitative research principles. The aim was not statistical generalization, but rather analytical depth and contextual understanding. This approach enabled the recruitment of participants with the greatest potential to provide relevant insights (29), maximized the richness of the data (30), and facilitated more productive discussions (31). The intentional selection process is characterized by a deliberate choice of participants, based on specific criteria, rather than random sampling (29). The participants were selected from a specific location and time frame, ensuring ease of access, and the sample was non-probabilistic in nature (27, 28).

Saturation was considered achieved when no new themes, subcategories, or insights emerged from the interviews. The final two interviews confirmed repetitive content, indicating conceptual redundancy. This concept aligns with Polit and Beck's (30) suggestion that saturation can be achieved with a relatively small number of cases. Minayo (32) further asserts that sample size in qualitative studies should be proportionate to the scope and complexity of the research. Given that qualitative studies typically involve a limited number of participants, the aim was to gain a detailed and in-depth understanding of the phenomenon under investigation (33). As it is impractical to study the entire population, the study focused on an accessible subset of the population (27).

Participant recruitment occurred during their consultation upon diagnosis of GD. At the conclusion of the consultation, pregnant women were invited to participate in the study. Those interested in joining the mindfulness group were asked to complete an informed consent form, which emphasized their right to withdraw from the study at any time without any impact on their care or their family's care.

During the recruitment phase, nine pregnant women who met the inclusion criteria were approached. One woman declined participation, while the remaining eight consented and completed the informed consent form. One participant withdrew during the study, and her data were excluded as per the informed consent guidelines. Consequently, the final sample consisted of nine pregnant women, aged 18 or older, attending the external obstetrics clinic and receiving diabetes care during the study period (January 17–26, 2024), who agreed to participate in the study.

### 2.2 Instruments and qualitative data analysis methods

To explore participants’ perceptions of mindfulness during pregnancy with gestational diabetes, the following guiding question was formulated: “*What are the perceptions of mindfulness among pregnant women with diabetes?”* This exploratory qualitative study was situated within a constructivist paradigm, which aims to understand how individuals make sense of their experiences in specific social and cultural contexts.

A semi-structured interview format was chosen as the primary data collection method due to its flexibility and ability to generate in-depth and spontaneous narratives. This method allowed participants to express personal experiences, emotions, and reflections in their own words, while still ensuring that core themes related to mindfulness were covered.

The interview guide was composed of 11 open-ended questions, structured into four thematic domains: (1) understanding of mindfulness, (2) effects on general well-being, (3) perceived physical and psychological benefits, and (4) relational impacts. The questions were developed based on the literature and refined through expert review to ensure clarity and appropriateness for the target population.

Prior to the interviews, participants received detailed information about the study's purpose and provided written informed consent. Interviews were conducted in a quiet, private setting to ensure comfort and confidentiality. Each session lasted approximately 20 min, was audio-recorded, and followed a conversational tone. The interviewer used probing questions and clarifications as needed, especially when participants were less familiar with mindfulness concepts. A non-judgmental and empathetic posture was maintained throughout, in line with ethical qualitative research standards.

All interviews were transcribed verbatim by the lead researcher and reviewed multiple times to ensure accuracy. Each transcript was assigned a code (E1–E8) to ensure anonymity and facilitate organization of the data. Field notes were taken to document contextual elements, non-verbal cues, and any relevant observations.

To promote methodological transparency, this study adhered to the Standards for Reporting Qualitative Research (SRQR) (26). The entire process, from interview planning and participant engagement to transcription was documented in a reflexive journal maintained by the researcher. This reflexivity helped monitor assumptions and minimize interpretive bias during data collection and analysis.

The data obtained were later analyzed using Bardin's content analysis method, which is further detailed in the subsequent section.

### 2.3 Qualitative data analysis

The qualitative data analysis was conducted using the content analysis model, as outlined by Bardin (34), which involves three phases: pre-analysis, material exploration, and the treatment of results, inference, and interpretation.

#### 2.3.1 Pre-analysis phase

The pre-analysis phase focuses on organizing and gaining a preliminary understanding of the content being studied. Following the completion of the interviews, the recordings were transcribed in their entirety to form the corpus for analysis. According to Bardin (34), this corpus must adhere to four key principles: exhaustiveness, representativeness, homogeneity, and relevance.

To ensure participant anonymity, the interviews were coded (E1–E8). A preliminary reading of the transcriptions was conducted to identify and delimit the material to be analyzed.

#### 2.3.2 Material exploration phase

In the material exploration phase, the content was coded, and units of meaning were extracted and enumerated. The extracted information was then coded, and the relevant textual segments, or “units of record,” were identified. These units were chosen based on their alignment with the study's research objectives and the characteristics of the analysis. Words that appeared frequently within the text were noted, as their significance increases with frequency, in line with Bardin's (34) approach.

The units of record correspond to specific textual segments within the data (34). Each subcategory was then constructed using these selected textual fragments.

#### 2.3.3 Treatment of results phase

The final phase, treatment of results, involves organizing the content to allow for the construction of meanings. This phase is critical for the interpretation of the findings and the identification of emerging patterns (34).

#### 2.3.4 Inductive and manual exploration

The coding process was conducted inductively. The researcher carefully read all transcripts, highlighted meaningful expressions (units of meaning), and grouped them into thematic clusters. For instance, phrases such as “feeling calmer” and “less stress” were grouped under the subcategory “Reduction of Stress and Anxiety.” Categories were then defined a posteriori to reflect emergent themes. This process allowed for a nuanced understanding of participants' perceptions.

While digital tools are available to facilitate content exploration, I chose to adopt an inductive and manual approach for data analysis. This method allows for a comprehensive evaluation and extraction of all possible information. Responses were segmented into units of record and grouped into various subcategories. As suggested by Bardin (34), semantic categorization was used to organize the data into thematic units, facilitating easier processing. The categories and subcategories were developed a posteriori, as no existing literature provided a framework for their establishment.

Given the lack of prior research on the perceptions of mindfulness among pregnant women, the results of this study and the categories that emerged from the analysis will be presented in subsequent sections.

#### 2.3.5 Ethical considerations and data management

In line with ethical standards, written consent was obtained from all participants through free and informed consent. This process ensured that participants were fully aware of the study's purpose, benefits, and their rights, including confidentiality, anonymity, and data privacy (35). Participants were also informed of their right to withdraw consent at any stage without any consequences for their care or participation in the study.

The information collected was used solely for the purposes of this study, maintaining the trust and confidentiality of the participants. All data were coded, processed, and analyzed by the researcher. Data from participant E6, who withdrew from the study, were excluded in accordance with the informed consent protocol.

Upon completion of the data transcription, all data were stored electronically and subsequently deleted after being fully processed. The study's data were stored on a personal computer protected by a unique password and permanently deleted once the data were no longer needed.

## 3 Results

### 3.1 Participant characteristics

In this study, a total of 7 participants were involved (*n* = 7), with ages ranging from 21 to 50 years (M = 32.43, SD = 9.519). The majority of participants were of Portuguese nationality, while the remaining participants came from countries where Portuguese is the primary language, which facilitated communication and understanding among the group.

Regarding educational background, 14% (*n* = 1) of the participants had not completed mandatory education. Most participants were employed in various fields, including commercial sectors and human and social sciences, such as law and human resources.

The majority of participants were married, with 29% (*n* = 2) being single but cohabiting with the fathers of their babies.

Concerning the planning and desire for the current pregnancy, 57% (*n* = 4) of the participants had planned their pregnancy. However, regardless of whether the pregnancy was planned, all participants (*n* = 7) expressed that the pregnancy was desired. Among the participants, 43% (*n* = 3) were in their third trimester and had been diagnosed with gestational diabetes (GD) following changes in their oral glucose tolerance test results. The remaining 57% (*n* = 4) had GD diagnosed due to altered fasting blood glucose levels during tests conducted in the late first trimester.

Prior experience with mindfulness varied among participants: some had previous exposure through interner, while others had no prior contact.

Regarding obstetric history, the majority of participants (57%, *n* = 4) were nulliparous, which, along with the average age of the participants, may help explain the occurrence of GD, as age is a known risk factor (36). Additionally, 14% (*n* = 1) of the participants reported having had two spontaneous abortions prior to their current pregnancy.

### 3.2 Perception of mindfulness

Through the content analysis of the interviews conducted with the 7 study participants, 1 with regard to the category Perception of Mindfulness, 7 subcategories emerged:

#### 3.2.1 Improvement in maternal-fetal well-being, physical contributions, psychological contributions, relational contributions and other contributions

In this sense, a table was created with the category, subcategories and registration units (Supplementary Table S1).

The findings provide new contributions to the area of perceptions regarding the technique of mindfulness.

#### 3.2.2 Improvement in maternal-fetal well-being

The participants highlighted the perception of *Improvement of Maternal-Fetal Well-being*, as mentioned in some studies (9, 11, 16). Through statements such as: “It really improves well-being” (E5); “It helps perhaps to have a greater sense of balance and well-being” (E4); “(..) it should positively influence the health of the pregnant woman and the baby” (E1); and “for the well-being of the baby, it already relieves us a lot” (E2).

Participants also revealed that mindfulness practice can contribute to a *Feeling of Peace and Happiness*, as it can “(..) bring more happiness and inner peace” (E5); “(..) we feel more happiness, more control in ourselves, overall, we feel more complete when the mind and body are aligned” (E3); and “Probably better understanding of emotions” (E7). Similar to what is described in the literature (37), participants recognized that mindfulness contributes to an increased sense of inner peace, although the studies have focused on students, not pregnant women. On the other hand, Crane et al. (38) reveals that mindfulness may contribute to greater emotional self-regulation, although there is a lack of studies linking mindfulness to sensations of peace, happiness, and emotional control for the population under study.

#### 3.2.3 Physical contributions

*Physical Contributions* emerged as another subcategory and are related both to the perception of *Reduction of Symptoms Associated with Pregnancy* and *Reduction of Symptoms Associated with Stress*.

Participants reported that mindfulness can reduce feelings of agitation and fatigue, symptoms associated with pregnancy. For instance, one participant stated: “It may help reduce activity and fatigue” (E1). Others found mindfulness useful for alleviating discomforts such as back pain and insomnia, with one participant noting: “It can have a very positive impact on physical well-being” (E3). Mindfulness also promoted a greater connection to bodily sensations: “It helps recognize what we’re feeling” (E4). Furthermore, it was noted that mindfulness could improve physical health, particularly in relation to breathing: “It even improves our health physically” (E2).

Regarding stress, participants described how mindfulness helped them recognize signs of anxiety, contributing to better emotional regulation through breathing: “I can tell by my breathing when I'm more anxious or calm” (E4). This aligns with research showing how mindfulness practices reduce sympathetic nervous system activity and lower respiratory rate, reducing symptoms associated with stress.

#### 3.2.4 Psychological contributions

The *Psychological Contributions* subcategory reveals the most significant contributions to the study, with numerous mentions of perceptions expressed by participants, and includes six indicators: *Increase in Maternal Self-Awareness*, *Reduction of Stress and Anxiety*, *Improvement of Self-Compassion and Compassion*, *Reduction of Depressive Symptoms*, *Stress Management Skills*, and *Mindfulness Skills*.

Increase in Maternal Self-Awareness was noted as mindfulness helped participants become more attuned to their bodies and pregnancy experience: “Being more aware of my body..” (E8). Recognizing the presence of the baby was also emphasized: “It can help increase awareness that there really is a baby here..” (E4), a sentiment previously identified by Santos et al. (16).

The Reduction of Stress and Anxiety (9–14) emerged as a key benefit, with participants noting that mindfulness helped alleviate stress and anxiety related to pregnancy: “To reduce stress and anxiety associated with pregnancy..” (E3).

Mindfulness also fostered Improvement of Self-Compassion and Compassion. Participants mentioned developing a more compassionate attitude toward themselves and others, with one stating: “Helps connect more with the body..” (E3) and another saying, “Not blaming myself for what I don't do” (E2). This aligns with Ferreira (11), who highlighted self-care as a key benefit of mindfulness.

Mindfulness was also recognized for Reduction of Depressive Symptoms in pregnancy (17, 39): “It may impact the reduction of depressive symptoms during pregnancy” (E8). Additionally, participants expressed that mindfulness helped improve Stress Management Skills: “A way to get through the most stressful situations..” (E2), and Mindfulness Skills: “A technique to learn to stop in our day-to-day life” (E5), enhancing their ability to be present and manage stress effectively.

#### 3.2.5 Relational contributions

The *Relational Contributions* subcategory includes three indicators: *Improvement of Emotional Relationships*, *Empathy*, and *Increased Maternal-Fetal Bond*.

Participants noted that mindfulness improved their Emotional Relationships, helping them to become more patient, understanding, and empathetic toward others. One participant stated: “It can help in the relationship with the husband and other children, to be more patient with them” (E1). Additionally, mindfulness fostered Empathy, as participants found it easier to relate to others' emotions: “We can put ourselves in others’ shoes..” (E5).

Mindfulness also helped in the Increased Maternal-Fetal Bond, with one participant noting: “It's really nice to feel this moment of connecting with the baby through mindfulness..” (E1), a sentiment that aligns with findings from Ferreira (11) and Vieten et al. (18).

#### 3.2.6 Other contributions

In addition to the main categories, several other contributions emerged regarding the practice of mindfulness. Although less frequently mentioned, these benefits are significant and provide further insight into its impact during pregnancy.

Mindfulness was frequently associated with relaxation, with participants highlighting its ability to promote both mental and physical tranquility: “It is a technique to relax the body and mind” (E1) and “Maybe using breathing to make the body more relaxed” (E3). The technique was perceived as helpful in reducing tension and achieving a calm state: “Anything that makes me more relaxed will be good” (E8).

Several participants acknowledged the benefits for childbirth, particularly in terms of pain relief and emotional regulation: “Breathing techniques to calm me down and reduce the pain during childbirth” (E8) and “It can probably help at the time of delivery” (E7).

Mindfulness was also associated with the development of mastery in the maternal role, with participants noting that the practice helped enhance their confidence and understanding of their new role: “It helps recognize the role of mother, because it is a role that has never been lived before” (E5) and “Recognizing and accepting experiences to feel more confident in being a mother” (E8).

## 4 Discussion

### 4.1 Interpretation of study findings and comparison with published evidence

Mindfulness practice during pregnancy has emerged as a promising intervention strategy; however, research specifically addressing its impact within high-risk pregnancy contexts, such as gestational diabetes (GD), remains limited. While substantial evidence supports the effectiveness of mindfulness in the general population, studies focused on its application in pregnancy—especially among women facing additional physiological and psychological challenges—are comparatively scarce (23). Notably, mindfulness-based approaches have demonstrated extensive benefits in non-perinatal contexts, suggesting potential for broader application (8).

The present findings, derived from participant narratives, highlight the importance of mindfulness in managing the emotional demands of pregnancy and fostering psychological equilibrium. This balance may positively influence both maternal and fetal health. Among the perceived benefits, psychological effects were the most consistently reported - echoing previous findings by Santos et al. (16). Conversely, physical and behavioral outcomes were less frequently mentioned, possibly reflecting the nature of the intervention delivery and limited exposure to the practice during the study period. Although mindfulness may appear conceptually simple, its practical application often requires sustained effort and guidance, particularly in sociocultural contexts marked by persistent cognitive overload and behavioral automatisms (40).

Sociocultural context of the participants, from Portuguese-speaking countries with limited exposure to contemplative practices like mindfulness. This shared linguistic and cultural background may have influenced their receptivity to the intervention and shaped their interpretations of its benefits.

Given that most reported benefits were psychological in nature, promoting mental health among pregnant women should be prioritized. A stable psychological state supports adaptive coping and resilience, especially during pregnancy - a period marked by physiological vulnerability and heightened stress reactivity (41). In addition to enhancing individual well-being, mindfulness may facilitate improvements in interpersonal dynamics, fostering more empathetic and patient interactions with partners, children, and family members. Furthermore, it contributes meaningfully to the maternal-fetal bond, improving a mother's sensitivity to the unborn child's needs (11).

Psychologically, the results underscore the value of mindfulness in mitigating stress and anxiety, fostering greater self-awareness and compassion, and potentially alleviating symptoms of depression (17, 39). Relationally, participants described improved empathy and a deeper sense of connection with others. Mindfulness was also associated with experiences of profound relaxation, tranquility, and serenity.

Regarding childbirth, participants perceived mindfulness as a helpful technique for pain management and preparation for labor, as well as for navigating the early stages of motherhood. The practice was seen as promoting a smoother transition into this new role, bolstering maternal confidence and presence.

A consistent theme across participant narratives was the link between mindfulness and improvements in both maternal and neonatal well-being. These improvements were frequently attributed to enhanced stress regulation, increased emotional awareness, and the development of self-compassion - factors that have been strongly associated with psychological resilience in the perinatal period (18). Reports of inner peace and emotional satisfaction suggest a broader impact on overall psychological functioning and well-being.

The data collected from this study highlights the multidimensional influence of mindfulness across key domains of pregnancy, including physical health, psychological resilience, interpersonal relationships, labor experiences, and maternal identity. Participants' descriptions reveal a coherent pattern of perceived benefits, which align closely with the existing literature. Mindfulness was repeatedly recognized as a versatile and accessible tool capable of supporting maternal mental health, enhancing physical comfort, and strengthening social bonds during pregnancy.

Although systematic coding and thematic saturation were achieved, the study did not incorporate validation mechanisms such as triangulation among researchers, member checking, or external auditing. This limitation is acknowledged and suggests directions for future research aiming to enhance the trustworthiness of qualitative findings.

In summary, these findings suggest that mindfulness may serve as a valuable adjunctive approach to comprehensive prenatal care, particularly in the context of high-risk pregnancies such as those affected by GD. Its capacity to promote physical, emotional, and relational well-being makes it a promising intervention to address the complex needs of this population.

### 4.2 Perspectives for clinical practice

The findings of this study indicate that mindfulness is perceived by pregnant women diagnosed with gestational diabetes (GD) as a valuable resource for enhancing overall well-being and facilitating a smoother transition into motherhood. Participants frequently reported positive effects of mindfulness, including stress reduction, increased self-confidence, and a greater sense of balance and control—all of which contribute to improved maternal adjustment (42).

Given these perceptions, mindfulness-based interventions hold promise for integration into nursing practice, particularly as a supportive strategy in the context of high-risk pregnancies. When facilitated by nurses, such interventions have demonstrated efficacy in lowering perceived stress and improving glycemic control among women with GD (42). These outcomes suggest that mindfulness may serve not only as a psychological support mechanism but also as a complementary modality in the physiological management of GD.

Furthermore, the comprehensive management of GD is increasingly centered on lifestyle-based interventions. Personalized nutrition counseling, structured physical activity programs, and behavioral support have been shown to significantly reduce the incidence of GD in at-risk populations and improve metabolic outcomes (43). Within this model, the role of nurses is fundamental. Nurses are uniquely positioned to support women with GD by promoting self-care practices focused on diet, exercise, emotional regulation, and medication adherence. Their person-centered and holistic approach has been associated with improved maternal and neonatal outcomes, while also enhancing the quality of life for women navigating the complexities of GD (44).

Integrating nurses into multidisciplinary care teams—as coordinators or case managers within lifestyle medicine frameworks—further strengthens the continuity and personalization of care. Evidence supports that such collaborative, proactive care models empower women with GD to make informed decisions and foster healthier behaviors during the perinatal period, ultimately improving outcomes for both mothers and their infants (45).

The study further suggests that mindfulness can be integrated into nursing practices to more effectively support pregnant women, especially in the context of high-risk pregnancies. Mindfulness, as highlighted by participants, contributes to a smoother transition into motherhood, reducing stress and enhancing emotional regulation. As the nursing role in managing high-risk pregnancies evolves, incorporating mindfulness as part of a broader care model could enhance maternal mental health, potentially influencing both physical and psychological aspects of pregnancy.

Mindfulness can be implemented by nurses through brief practices during prenatal consultations, such as 5-minute breathing exercises or body scans. Group antenatal classes could also include mindfulness sessions to high-risk pregnancies. These practices, when led by trained nurses, can support psychological well-being and improve glycemic control. Future guidelines should consider incorporating mindfulness in standard antenatal care pathways.

The multidisciplinary and multidimensional management of diabetes, particularly in high-risk populations such as pregnant women with GD, remains an important area for exploration. Nurses, through their role in diabetes care, are pivotal in promoting individualized lifestyle changes, such as healthy eating, exercise, stress management, and medication adherence. This approach not only helps manage GD but also improves quality of life, reducing the risk of complications during pregnancy and postpartum.

In conclusion, the integration of mindfulness into nursing care presents a promising approach for improving the well-being of pregnant women with GD. By adopting a holistic, person-centered care model, nurses can offer continuous support, empowering women to make informed choices and fostering better health outcomes for both mother and baby. Future research is needed to further explore the broader implications of mindfulness-based interventions in high-risk pregnancy care, with a focus on expanding the evidence base to support its integration into clinical practice.

### 4.3 Limitations

During the research, several limitations impacted the completion of the study. One significant limitation was the insufficient number of existing studies on the topic, which hindered the provision of robust theoretical support for the theme.

The limited number of pregnant women attending consultations also reduced the pool of potential participants, further constraining the study's sample size. Additionally, since this was a descriptive study aimed at capturing the characteristics of a specific reality, its qualitative nature means that the results cannot be generalized to broader populations.

The study also did not include member checking, peer debriefing, or an audit trail, all of which are commonly used strategies to enhance the trustworthiness of qualitative research. Although manual coding and systematic content analysis were conducted, the lack of these additional validation techniques is recognized as a limitation. Future studies are encouraged to incorporate these methods to further enhance the credibility and reliability of their findings.

## 5 Conclusions

This study explored the perceptions of mindfulness among pregnant women diagnosed with gestational diabetes (GD), aiming to understand its perceived impact during this high-risk period. The qualitative analysis revealed mindfulness as a multifaceted intervention that provides benefits across physical, psychological, emotional, and relational domains.

Participants identified mindfulness as a valuable resource for enhancing both maternal and fetal well-being, particularly through emotional regulation, increased self-awareness, and inner calm. The most frequently reported benefits were psychological, including reductions in stress and anxiety, enhanced self-compassion, and improved coping strategies. Physically, participants associated mindfulness with reduced discomfort and greater bodily awareness. Relationally, it was perceived as fostering empathy, strengthening interpersonal relationships, and promoting the maternal-fetal bond. Additionally, mindfulness contributed to a sense of empowerment by supporting relaxation, preparing for labor, and easing the transition to motherhood.

These findings suggest that mindfulness is a relevant, low-cost, and accessible intervention in prenatal care, especially for women with GD, who often face heightened psychological and physical challenges. Although limited by a small sample size and the qualitative nature of the research, which restricts generalizability, this study highlights mindfulness' potential to enhance holistic, patient-centered nursing care and foster a more conscious, connected pregnancy experience.

Future research should involve larger, more diverse populations while evaluating the effectiveness of structured mindfulness-based interventions throughout all stages of pregnancy, particularly in women with high-risk conditions like GD. Furthermore, as studies to date have focused primarily on the second trimester, further investigation is needed to fill knowledge gaps across the entire perinatal period.

Lastly, future studies should consider including family members and partners in mindfulness practices, as their participation may further enhance maternal well-being and comfort. Continued research is crucial to developing robust, evidence-informed strategies that improve nursing care and support a safe, empowered, and meaningful transition into motherhood.

## Data Availability

The original contributions presented in the study are included in the article/Supplementary Material, further inquiries can be directed to the corresponding author.
