# Supplementary material for: Perceptions of mindfulness to pregnant women with gestational diabetes: an exploratory qualitative Portuguese study
Source: Front Glob Womens Health. 2025 Sep 11;6:1558231. doi: 10.3389/fgwh.2025.1558231 (PMC12460244; doi:10.3389/fgwh.2025.1558231)
Supplement: Supplementary file 1 [file Table1.docx]

Supplementary Material

# Supplementary Data

**Table 1. Subcategories, and Indicators related to the category *Perception of Mindfulness***

| **CATEGORY** | **SUBCATEGORY** | **INDICATORS** |
| --- | --- | --- |
| *Perception of Mindfulness* | *Improvement in Maternal-Fetal Well-being* | Improvement of maternal and baby health well-being |
|  |  | Feeling of peace and happiness |
|  | *Physical Contributions* | Reduction of symptoms related to pregnancy |
|  |  | Reduction of symptoms related to stress |
|  | *Psychological Contributions* | Increased maternal self-awareness |
|  |  | Reduction of stress and anxiety |
|  |  | Improvement of self-compassion and compassion |
|  |  | Reduction of depressive symptoms |
|  |  | Stress management skills |
|  |  | Mindfulness skills |
|  | *Relational Contributions* | Improvement in emotional relationships |
|  |  | Empathy |
|  |  | Increased maternal-fetal bond |
|  | *Other Contributions* | Relaxation |
|  |  | Benefits for childbirth |
|  |  | Mastery of the role of mother |

# Appendix – SRQR Table (Standards for Reporting Qualitative Research)

This table outlines the 21 SRQR items (O’Brien et al., 2014), with their corresponding application in the present qualitative study.

| **No.** | **Topic** | **SRQR Item** | **Application in the Study** |
| --- | --- | --- | --- |
| S1 | Title | Concise description of the study and its qualitative nature. | ✔ Yes, identified as exploratory qualitative. |
| S2 | Abstract | Structured summary with background, methods, results, and conclusions. | ✔ Yes. |
| S3 | Problem formulation | Description of the phenomenon and relevant literature. | ✔ Yes, provided in the introduction. |
| S4 | Purpose or research question | Study objectives or research questions. | ✔ Yes, clearly stated. |
| S5 | Qualitative approach and research paradigm | Type of qualitative approach and epistemological paradigm. | ✔ Yes. |
| S6 | Researcher characteristics and reflexivity | Attributes and role of the researcher, and influence on the research. | ✖ Include researcher background, relationship with participants, and reflexivity about potential biases. |
| S7 | Context | Study setting and relevant contextual factors. | ✔ Yes, hospital and obstetric setting described. |
| S8 | Sampling strategy | How and why participants were selected. | ✔ Yes, purposive sampling explained. |
| S9 | Ethical issues | Ethics approval, consent, confidentiality. | ✔ Yes, well described. |
| S10 | Data collection methods | Types and processes of data collection. | ✔ Yes, semi-structured interviews conducted. |
| S11 | Instruments and technologies | Interview guides and recording devices. | ✔ Yes, guide and audio recording mentioned. |
| S12 | Units of study | Number and characteristics of participants. | ✔ Yes, n=7, with detailed profile. |
| S13 | Data processing | Transcription, anonymization, data management. | ✔ Yes, detailed transcription process described. |
| S14 | Data analysis | Coding and inference methods. | ✔ Yes, Bardin’s content analysis applied. |
| S15 | Techniques to enhance trustworthiness | Validation methods like member checking, triangulation. | Partially – manual coding mentioned |
| S16 | Synthesis and interpretation | Main findings and themes. | ✔ Yes, categories and subcategories well presented. |
| S17 | Links to empirical data | Quotations or data supporting results. | ✔ Yes, interview excerpts provided. |
| S18 | Integration with prior work | Comparison with existing literature. | ✔ Yes, literature integrated in discussion. |
| S19 | Limitations | Study limitations and trustworthiness issues. | ✔ Yes, addressed. |
| S20 | Conflicts of interest | Disclosure of any influences. | ✔ Yes, declared. |
| S21 | Funding | Funding sources and roles. | ✔ Yes, stated as no financial support. |

Interview script

|  | Issues | Evaluation Indicators |
| --- | --- | --- |
| 1 | What do you consider mindfulness to be *?* | - Explore the definition of *mindfulness* for each pregnant woman |
| 2 | Have you practiced the *mindfulness technique?* | - Explore past and present experiences of pregnant women  - Reporting personal experiences or observations of improvements in well-being during pregnancy. |
| 3 | Do you believe that the practice of *mindfulness* can have an impact on the well-being of pregnant women? | Impact on Overall Well-Being and Improvement in Health  - Expression of positive beliefs about the benefits of *mindfulness* on overall well-being.  - Reporting perceptions about how *mindfulness* can have positive impacts on the overall health of pregnant women.  - Identification of benefited health areas. |
| 4 | How do you see the possible contribution of *mindfulness* to physical well-being during pregnancy? | Contribution to Physical Well-Being  -Identification of specific perceptions by which *mindfulness* can contribute to physical well-being.  - Description of changes in physical health associated with *mindfulness practice*. |
| 5 | What is your perspective on the influence of *mindfulness* on the risk of having a preterm birth? | Reducing the Risk of Preterm Birth  - Expression of opinions on the relationship between *mindfulness* and the reduction of the risk of preterm birth. |
| 6 | How do you see the possible contribution of *mindfulness* to psychological well-being during pregnancy? | Contribution to Psychological Well-Being  - Identification of specific perceptions by which *mindfulness* can contribute to psychological well-being.  - Evaluation of the perceived effectiveness of *mindfulness* in reducing stress during pregnancy.  - Discussion of specific situations in which *mindfulness* will be useful for managing stress.  - Evaluation of the perceived effectiveness of *mindfulness* in reducing anxiety during pregnancy.  - Discussion about specific situations in which mindfulness will be useful to manage anxiety. |
| 7 | To what extent can *mindfulness* influence depressive symptoms during pregnancy? | Efficacy in Reducing Depressive Symptoms and Alternative to Drug Treatment  - Expression of perceptions about how *mindfulness* can influence the reduction of depressive symptoms.  - Identification of perspectives on the validity of *mindfulness* as an alternative to drug treatment.  - Discussion on the feasibility of *mindfulness* as a treatment for depression in pregnant women. |
| 8 | In your opinion, how can *mindfulness* influence maternal self-awareness? | Increased Maternal Self-Awareness and Significant Increases in Mindfulness Skills  - Discussion of how *mindfulness* can influence the increase in maternal self-awareness.  - Identification of behaviors or changes associated with a greater understanding of herself as a mother.  - Discussion of how *mindfulness* can strengthen the capacity for attention to the present during pregnancy. |
| 9 | What is your perspective on mindfulness's contribution to self-compassion and compassion? | Increased Self-Compassion and Compassion  - Expression of perceptions about how *mindfulness* contributes to increased maternal self-compassion and compassion. |
| 10 | In your opinion, to what extent can *mindfulness* contribute to relational well-being during pregnancy? | Contribution to Relational Well-Being  - Identification of ways in which *mindfulness* can contribute to relational well-being during pregnancy.  - Discussion about how *mindfulness* practices can impact interpersonal relationships. |
| 11 | What is your perspective on the influence of *mindfulness* on the bond between mother and baby? | Increased maternal-fetal bonding  - Discussion of the perceived mechanisms behind this increase in calling. |

Table of Content Analysis of the Interviews

| **Categories** | Subcategories | *Indicators* | *Description Registration Units* | Frequency |
| --- | --- | --- | --- | --- |
| **Insight into mindfulness** | **Improvement in Maternal-Fetal Well-being** | Improved well-being in maternal health and baby health | *"(...) it should positively influence the health of the pregnant woman and the baby." (E1)*  *"Yes, it should improve well-being." (E3)*  *“(…) it must have good applicability to all areas of your life (...)" (E4)*  *“(…) it greatly improves well-being." (E5)*  *"Yes, it should have a positive impact on life and the sense of well-being, both physical and psychological, which are always interconnected, isn't it..." (E7)*  *"Yes, of course, it must be very positive, a very good experience to have during pregnancy (...)" (E8)*  *"It helps maybe to have a greater sense of balance and well-being." (E4)*  *"our mother's physical and psychological well-being will influence the baby in several and varied ways" (E2)*  *"it will contribute to our well-being and at all levels I think I have only good things to add" (E2)*  *"we are trying and doing something for our well-being" (E2)*  *"for the baby's well-being it is already something that will relieve us a lot" (E2)* | 11 |
|  |  | Feeling of peace and happiness | *"(...) bring more happiness and more inner peace." (E5)*  *“(…) find moments of inner peace (...)" (E5)*  *"(...) to find more peace of mind (...)" (E8)*  *"(...) The mind is in the order of these questions that happen physically." (E2)*  *"(...) we feel more happiness, more control in ourselves, in general we feel that we are more complete when the mind and body are aligned" (E3)*  *"Anything that can help promote a state of mental tranquility looks positive in pregnancy." (E3)*  *"Without a doubt, to feel more in a good mood (...)" (E3)*  *"(...) to ward off negative thoughts (...)" (E2)*  *“(…) to control emotions more." (E3)*  *"Pregnancy can be a very challenging time, and practicing techniques to calm the mind and deal with mood can be very good." (E4)*  *"(...) to calm the mind (...) valuable for psychological well-being." (E5)*  *"Probably to understand emotions better (...)"(E7)* | 12 |
|  | **Physical Contributions** | Decrease in symptoms associated with pregnancy | *"It can contribute to being less accelerated, I'm always on 220V, and I get more tired so it can help reduce activity and tiredness." (E1)*  *"(...) which can have a very positive impact on physical well-being. It can be useful to deal with some discomforts, such as back pain or insomnia." (E3)*  *"These techniques can be very good for tuning in to the sensations of our body and recognizing more what we are feeling." (E4)*  *"I see that it can contribute in a positive way (...)" (E8)*  *“(…) related to breathing (...) even improve our health here in even physical terms (...)" (E2)* | 5 |
|  |  | Decrease in symptoms associated with stress | *“(…) I can tell by breathing, by the way I can see some triggers in me, in my body when I'm more anxious or calm." (E4)*  *"(...) that we walk less accelerated, less worried and this also has physical implications in breathing, in the feeling of tightness of the heart and in that feeling of almost burning the throat when I am stressed." (…)” (E5)*  *"In fact, psychological well-being influences our entire body, including the physical, nervousness gives that body tremor and that feeling of a heavy stomach, so it can undoubtedly help in this management of the sensations caused by the nerves." (E2)* | 3 |
|  | **Psychological Contributions** | Increased maternal self-awareness | *“(…) to be more conscious about my body (...)" (E8)*  *“(…) it is to be more aware of what we do pregnant women (...) (E1)*  *“(…) to give material here so that we can be more aware of what we are experiencing, what we are going through, what may or may not happen (...)" (E2)*  *"Motherhood is a sea of complexity, so being more aware of the world around me seems to me to be a positive attitude." (E3)*  *"I believe it could increase your self-awareness. Helping to make more conscious decisions, yes" (E4)*  *"It involves being more aware (...) can help to have this awareness of what we have become" (E5)*  *“(…) help us to be more aware of ourselves and others (...)" (E2)*  *"I think it should make us more condescending (...) to live with more awareness of what is going on." (E3)*  *"It can help us to be more aware (...)" (E5)*  *“(…) the fact that we are aware (...)" (E2)*  *“(…) it can help raise awareness that there really is a baby here (...)" (E4)*  *“(…) perceiving that we are more aware of ourselves (...)" (E4)* | 12 |
|  |  | Reduction of stress and anxiety | *"(...) help reduce stress for sure." (E4)*  *“(…) to reduce the stress and anxiety associated with pregnancy (...)" (E3)*  *"It can help pregnant women better cope with emotional stress and anxiety." (E4)*  *“(…) situations in which I feel more stressed." (E7)*  *"(...) I get less stress (...)" (E1)*  *“(…) reduce all this anxiety, because I want everything to go for the best. It's impossible to take away all the anxiety, but who knows if it can help reduce it." (E1)*  *"(...) help reduce anxiety (...)" (E2)*  *“(…) maybe it will reduce anxiety (...) (E5)"* | 8 |
|  |  | Improved self-compassion and compassion | *“(…) to like myself more (...)" (E5)*  *"It helps to connect more with the body (...)" (E3)*  *“(…) and enjoying taking care of myself more, I spent all the pregnancies taking care of the baby and in that one I really have to take care of myself more, in what I eat, in what I do." (E1)*  *"Do you know what I think is really important for motherhood? Learning to like myself was not an easy path to acceptance because I really liked my body, but I have to accept that it is for the greater good." (E3)*  *"(...) to understand the needs and to take better care of myself, to see myself as a priority" (E7)*  *"Charge myself less for what I don't do" (E2)*  *"It may be that greater compassion for others is developed" (E3)*  *“(…) to like ourselves more (...)" (E4)*  *"It should help us to like ourselves more (...) to have that compassion." (E5)*  *"Because it can help to look more at myself and not so much at the problems that come, not to put ourselves in the background, to be more concerned with myself, more affectionate, to try to understand more my feelings and all these internal processes" (E8)*  *“(…) I think it helps more in the relationship with myself (...)" (E3)* | 11 |
|  |  | Reduction of depressive symptoms | *“(…) which can indeed have an influence on pregnancy, but it can be even more important after childbirth. (E1)*  *"Well, if we live a calmer pregnancy, without a doubt this will influence, for example, an implication in postpartum depression, I have already been told about postpartum depressions in the preparation course I am doing and it really is stress has a lot of influence". (E2)*  *"Yes, without a doubt it can help reduce the probability of having these symptoms (...)" (E3)*  *“(…) There are some triggers and a way to drive, and that made me stop taking medicine." (E4)*  *"(...) it can be useful for other pregnant women who have depression (...)" (E5)*  *"(...) It also helps not to have these symptoms." (E7)*  *"(...) may have an impact on reducing depressive symptoms in pregnancy." (E8)*  *“(…)sometimes the mind is capable of curing many things, when I studied, I knew that there were people who had an HIV diagnosis, who managed to reverse it with therapy, this is something that is fantastic (...) results and most patients were significantly superior when people had psychological support (...)" (E2)* | 8 |
|  |  | Stress management skills | *“ (…) good strategy to use in situations that may happen now during pregnancy (...)" (E3)*  *“ (…) if it doesn't help to find a way to overcome the most stressful situations (...)" (E2)*  *“(…) also in the way we are probably going to manage stress (...)" (E7)*  *"(...) to deal with worries (...)" (E8)*  *"(...) to deal with stress (...)" (E8)*  *"(...) maybe in calming down, thinking and directing our thinking towards the solution and not so much towards the problems." (E2)* | 6 |
|  |  | Mindfulness skills | *“(…) a technique for us to learn to stop in our day-to-day running by (E5)*  *"(...) with our attention and that, in some way (...)" (E2)*  *“(…) I believe mindfulness can help them feel more centered (...)" (E3)*  *"By being more aware of what is happening at the moment (...) (E5)"*  *"Then it helps to concentrate more on the present (...)" (E8)*  *“(…) we have to, in a way, be focused to be able to interact with others in a positive way (...)" (E2)*  *“(…) if we are focused (...)" (E2)*  *"It can help more to focus on the moment (...)"(E8)* | 8 |
|  | **Relational Contributions** | Improvement in affective relationships | *“(…) it must have an influence on the physical, psychological and even relationship parts (...)" (E4)*  *"Yes, it can help in the relationship with the husband and with the other children, to be more patient with them." (E1)*  *“(…) Not being stressed, anxious, I will be able to have a positive presence and talk better, be willing to listen to other people. I think that if the person is more relieved, if they are calmer and if they even have techniques that they can learn to relate to others, even when others present some kind of issues, it is also not that we have these skills, I think it will be very good because it produces more harmonious and positive relationships." (E2)*  *"I think it can help in relationships with others (...)" (E3)*  *"I think that when we get too stressed, we end up going against the person who is closest to us and you learn not to do this to the person, who just wants to help. Nobody likes not to be treated very well."" (E4)*  *“(…) what we talk about with other people and how we treat them, because in pregnancy, at least I speak for myself, I say everything I think, it can help to filter this information, think about it before speaking, so as not to hurt people" (E5)*  *"Yes, I also think it is capable of helping" (E7)*  *“(…) I give this very much this example which is: with the arrival of a newborn the relationship is totally different, it will not be the same as it already was and then, for example, my husband arrives at night tired and does not realize that at home I had a lot of work but I also have to realize that he also had his work and so being able to put myself in the place of the other helps a lot to maintain a relationship" (E8)* |  |
|  |  | Empathy | *“(…) more aware of their lives and can pay more attention to those around them" (E3)*  *“(…) look at the other in a different way. I believe that this is enough, I'm having more sensitivity, isn't it? to ourselves and to others for sure" (E4)*  *“(…) to be able to put ourselves in the place of others (...)" (E5)*  *“(…) beneficial to feel more pity for the other, not to see only my problems as mine, to share that pain a little." (E7)*  *"We put a lot in the place of the other (...)" (E8)* |  |
|  |  | Increased maternal-fetal bonding | *“(…) maybe with my baby, creating a connection, that sometimes I even forget that I'm pregnant as I don't feel it move (...) To have a connection earlier, even if I don't feel it. And then it should help when he is born to maintain that connection that I believe is stronger (...)" (E3)*  *"It should help a lot to increase this connection." (E1)*  *"It certainly has some positive influence (...)" (E5)*  *“(…) who knows if it may not also increase." (E7)*  *"Forgetting the world and thinking that it's just me and my baby, sometimes it's not easy to make this wall for the world, but it would be so good, to create just a space for me and him". (E8)* |  |
|  | **Other Contributions** | Relaxation | *“(…) a technique to relax the body and mind." (E1)*  *“(…) these issues because relaxation itself is so technical (...)" (E2)*  *"Maybe use breathing to make the body more relaxed." (E3)*  *“(…) A technique of relaxing the body, to have more tranquility, to have a more open mind is out there." (E4)*  *"technique of relaxing, of learning to breathe" (E5)*  *“(…) relaxation technique (...)" (E7)*  *“(…) Anything that makes me more relaxed will be good." (E8)*  *“(…) relaxed (...)" (E1)*  *“(…) techniques of reorganization of restructuring of relaxation thinking (...)" (E2)*  *“(…) form of relaxation (...)" (E4)*  *"It's possible that because we become more relaxed (...)" (E2)* | 11 |
|  |  | Benefits for childbirth | *“(…) breathing techniques to make me calmer and reduce the pain in childbirth (...)" (E8)*  *“(…) very useful in pregnancy and childbirth (...)" (E2)*  *“(…) It can probably help at the time of delivery." (E7)* | 3 |
|  |  | Mastery of the role of mother | *“(…) Equip ourselves here with skills to face adversities and issues that may be inherent to this phase, but that we have no idea about. We are very overwhelmed thinking about many other things and we don't focus on calming down, thinking and directing our thinking towards the solution and not so much towards the problems." (E2)*  *"(...) to help better recognize the role of mother, because it is a role that has never been lived before (...)" (E5)*  *“(…) recognize and accept experiences to feel more confident in being a mother, which is not easy, at first there are many opinions from others." (E8)* | 3 |
